# Pre- and Postsynaptic Dopamine SPECT in Idiopathic Parkinsonian Diseases: A Follow-Up Study

**DOI:** 10.1155/2013/143532

**Published:** 2013-09-19

**Authors:** Susanna Jakobson Mo, Jan Linder, Lars Forsgren, Henrik Holmberg, Anne Larsson, Katrine Riklund

**Affiliations:** ^1^Department of Radiation Sciences, Diagnostic Radiology, Umeå University, 90187 Umeå, Sweden; ^2^Department of Pharmacology and Clinical Neuroscience, Neurology, Umeå University, 90187 Umeå, Sweden; ^3^Department of Statistics, Umeå School of Business and Economics, Umeå University, 90187 Umeå, Sweden; ^4^Department of Radiation Sciences, Radiation Physics, Umeå University, 90187 Umeå, Sweden

## Abstract

We prospectively evaluated the diagnostic contribution of ^123^I-FP-Cit (DAT) and ^123^I-IBZM (IBZM) SPECT in 29 patients with Parkinson's disease (PD) (74.4 ± 4.2 years) and 28 patients with atypical parkinsonian diseases (APD) (74.3 ± 9.2 years). Twelve had multiple system atrophy (MSA) and 16 progressive supranuclear palsy (PSP). Sixteen age-matched healthy controls (HC) were included. DAT and IBZM SPECTs were made at baseline and after 1 year in all PD patients and in 20 (DAT) and 18 (IBZM) of the APD patients, and after 3 years in 22 (DAT) and 17 (IBZM) of the PD patients and in 10 (DAT) and 10 (IBZM) of the APD patients. The relative DAT uptake decrease was faster in PD and PSP than in HC and MSA. In PSP the DAT uptake was lower than in MSA after 1 year but not after 3 years. Baseline IBZM uptake was not significantly different between patients and HC or between PD and APD. One year after initiated dopaminergic treatment the mean IBZM uptake in the MSA patients remained high compared to PSP and after 3 years compared to PD, PSP, and HC. Thus, the pattern of uptake of these ligands over time may be of value in discriminating between these diagnoses.

## 1. Introduction

The atypical parkinsonian diseases (APD) multiple system atrophy (MSA) and progressive supranuclear palsy (PSP) are less common than Parkinson's disease (PD), but since APD patients share the parkinsonian symptoms, the clinical distinction between PD and APD may be difficult in early phases [1, 2]. The clinical diagnostic accuracy can be improved if specialists in movement disorders make the diagnosis [3], but still the accurate diagnosis may take time to unveil. In PD, MSA, and PSP there is a degeneration of dopamine neurons in substantia nigra, leading to depletion of dopamine in the nigrostriatal system [4]. The use of dopamine transporter (DAT) ligands, such as ^123^I-FP-Cit (N-*ω*-fluoropropyl-2-*β*-carbomethoxy-3*β*-(4-[123I]iodophenyl)nortropane), ^123^I Ioflupane, (GE Healthcare B.V., Eindhoven, The Netherlands), to examine the integrity of the nigrostriatal dopamine function with imaging techniques, such as single photon emission computed tomography (SPECT), have shown high sensitivity in idiopathic parkinsonian diseases [5, 6] but a low discriminative power in separating PD from APD [7]. SPECT imaging with a postsynaptic dopamine D2/D3 receptor ligand, such as ^123^I-IBZM (S)-2-hydroxy-3-[123I]iodo-6-methoxy-*N*-[(1-ethyl-2-pyrrolidinyl)methyl]-benzamide, ^123^I Iolopride, (GE Healthcare B.V., Eindhoven, The Netherlands), [8] has been used to discriminate APD from PD based on the histopathological differences in degeneration in the striate [4, 9–12]. However, the discriminative power is still under debate [13, 14]. The additional diagnostic value of ^123^I-IBZM SPECT in very early APD is sparsely studied, prospective studies are few [10, 15, 16], and to our knowledge, no prospective studies combining these two ligands have been performed in PD and APD patients in the early phase of the disease. 

Pre- and postsynaptic dopamine SPECTs of prospectively followed and age-matched patients with similar duration of PD, MSA, and PSP were evaluated. The diagnoses were assessed after several years of clinical followup and were entirely based on established clinical criteria. Additionally, the SPECT uptake at baseline and after 3 years in a group of age-matched healthy controls (HC) was used for comparison. The uptake patterns and change over time were measured using a ROI method. The aim of this study was to investigate if, or when in the course of the disease, the addition of ^123^I-FP-Cit (DAT) SPECT and ^123^I-IBZM (IBZM) SPECT may be of use in the diagnostic workup of early idiopathic parkinsonian diseases.

## 2. Materials and Methods

### 2.1. Study Population

The study population in the present study was selected from a large, on-going longitudinal clinical research project (main clinical project; inclusion time January 1, 2004–April 30, 2009) and imaging study (SPECT substudy) on idiopathic parkinsonism [17], in which the patients and HC are followedup clinically and with a number of auxiliary tests as well as structural MRI for up to 8 years. 

Twenty-eight patients that fulfilled the established clinical criteria for APD after clinical followup in the above mentioned research project were included in the present study. Additionally, 29 age-matched PD patients fulfilling the criteria for clinical definite PD and 16 age-matched HCs were selected from the same research project. 

In the present study, the DAT and IBZM SPECTs were done on a hybrid gamma-camera system, an Infinia Hawkeye (General Electric, Milwaukee, WI, USA) at baseline and at sequential timepoints up to 3 years from inclusion. Gender, age at SPECT and age at symptoms' onset are shown in Table 1. The clinical survey time (reflecting the maturity of the clinical diagnoses) in the patients and the HCs is shown in Table 2.

The regional ethics committee in Västerbotten, the local radiation safety committee, and the Swedish medical products agency approved the study. Recruited patients and HC were included after written and oral consent according to the declaration of World medical Association Declaration of Helsinki.

#### 2.1.1. Multiple System Atrophy and Progressive Supranuclear Palsy

For this paper, patients having done at least one DAT SPECT and/or IBZM SPECT on the above mentioned hybrid gamma-camera were selected. In the main clinical project, a total of 15 patients fulfilled the diagnostic criteria for MSA [18]. Of these, 2 patients did not participate in the SPECT substudy and one MSA patient had only a single baseline SPECT that was made with another type of SPECT camera without further SPECT followup and was therefore not selected for the present study. 

Sixteen patients in the main clinical project fulfilled the diagnostic criteria for PSP [19] after up to 5 years of clinical followup, and all of them participated in the SPECT substudy. The selection for the present study thus resulted in 12 MSA patients and all 16 of the initially included PSP patients.

Several of the APD patients additionally fulfilled the UK Parkinson's Disease Society Brain Bank (UK PDSBB) diagnostic criteria for PD [20]. The numbers of SPECTs, gender, diagnoses, and motor subtypes are presented in Table 3. Data on the number of SPECTs in MSA and PSP patients with corresponding clinical data per followup timepoints is presented in Table 4.

#### 2.1.2. Parkinson's Disease

PD patients were selected if (i) the diagnostic criteria of clinically definitive PD [20] were fulfilled and (ii) at least a baseline and one follow-up SPECT was done on the hybrid gamma-camera and (iii) age at baseline corresponded to the age span of the APD patients (mean ± 2SD). All selected patients had a pathological DAT SPECT, and no patient who had undergone DBS surgery was included. This resulted in a group of 29 patients (m/f 18/11). The number of SPECTs in the PD group with corresponding clinical data is presented in Table 4. Twenty-three of the 29 PD patients (m/f 15/7) had a SPECT after 3 years. One DAT SPECT at year 3 was excluded due to poor image quality. Seventeen of the originally selected PD patients were examined with IBZM SPECT at the third year followup. 

#### 2.1.3. Healthy Controls

HCs with ages within the age range of the patients and with a clinical followup time of 5 years were selected. These had at least one DAT SPECT or IBZM SPECT at baseline as well as follow-up SPECTs after 3 years. The selection resulted in a group of 16 individuals (m/f 7/9). HCs were clinically evaluated at baseline and followup according to a detailed neurological and physical protocol. None of the HCs had any clinical signs of parkinsonism or other clinical signs of neurological disease at baseline or followup. The number of HC and age at SPECT is shown in Table 1.

#### 2.1.4. Clinical Parameters in Patients

**Figure 1:**
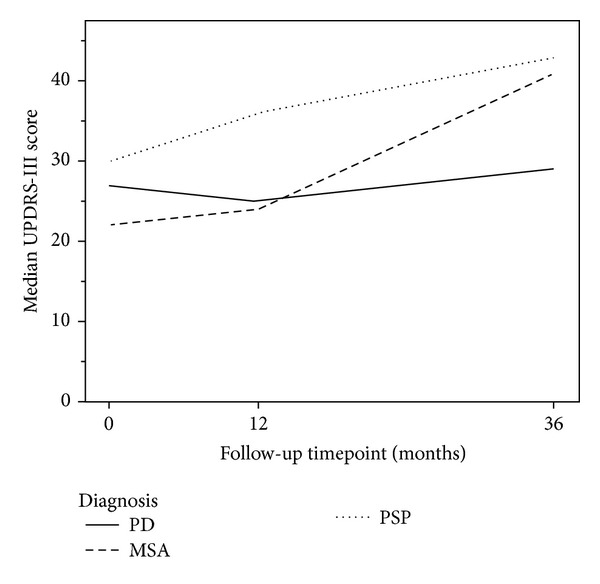
Median UPDRS-III sub score in the PD, MSA and PSP patients at baseline, and after 1 and 3 years of follow-up. *MSA: Multiple System Atrophy, PSP: Progressive Supranuclear Palsy; PD: Parkinson's Disease. *

The clinical data on the selected patients in this study are presented in Table 4. PSP patients had higher Unified Parkinson's Disease Rating Scale—motor score (UPDRS-III), higher Hoehn and Yahr (H&Y) stage, and lower score for activities of daily living (ADL) compared to PD at both follow-up occasions (i.e., 1 and 3 years). The MSA patients had higher UPDRS-III score, H&Y stage and lower ADL score compared to PD at the third year follow-up occasion. The relative change in UPDRS-III scores between baseline and the first followup was significantly different in MSA and PSP patients compared to PD. PD patients tended to decrease in UPDRS-III scores (signifying a motor improvement) and APD patients tended to increase (motor deterioration) during the survey, as can be seen in Figure 1. The dopaminergic treatment as expressed by L-Dopa equivalent dose (LED) index [21] was not significantly different between the groups as displayed in Table 4.

#### 2.1.5. Drop-Off

As shown in Table 3, five of the MSA patients and 3 of the PSP patients were examined with another type of SPECT camera at baseline, and 2 of the MSA patients were reexamined with that camera also after 1 year. Since the semi quantitative measurements derived from these two SPECT cameras are not directly comparable, examinations done on the other gamma-camera were not included for analysis in the present study. 

Drop-off in the APD group was attributable to inability to sustain temporary pharmacological withdrawal, disease severity, (making it too demanding to lie still in the SPECT camera) refusal, or death. There were no statistical differences between the APD patients with and without SPECT at the third year follow-up timepoint with respect to disease severity (as measured with UPDRS III points and H&Y score), neither age at onset or age at inclusion. The APD patients remaining in the SPECT group at followup (i.e., individuals with either DAT- or IBZM SPECT or both, *n* = 12) had higher LED index, mean 554.2 (95% CI: 394–715) compared to those still clinically followed patients without SPECT (available clinical data for 13 cases), mean 319.2 (95% CI: 186–452), *P* < 0.05, and they also had a longer mean clinical follow-up time, 5.0 (4.2–5.8) years compared to 3.5 (2.8–4.2) years, *P* < 0.005. In the PD group there were no statistically significant differences in age at onset, age at inclusion, clinical follow-up time, disease severity, or LED index between patients with and without SPECT at the third year follow-up timepoint.

#### 2.1.6. Presenting Motor Subtypes and Laterality

In PD, symptoms are typically asymmetrical in the early phase of the disease, reflecting an asymmetrical degree of dopamine degeneration between hemispheres, with the most affected side in the brain contralateral to the most affected limb or body-half. Still, symptoms at presentation were symmetrical or without any specific laterality (e.g., global bradykinesia as the main presenting symptom) in half of the patients in this study. The majority of the APD patients presented with postural instability and gait difficulty (PIGD) motor subtype at baseline without specific laterality (Table 5(a)). The side with the lowest DAT uptake in the putamen at baseline was considered to be the most affected side in all patients. If subsequent SPECT follow-up examinations exhibited a side-shift of the lowest putaminal uptake, the laterality was judged inversed if the side-to side difference exceeded 5%. This was the case in 3 APD patients and 2 PD patients at the first year follow-up SPECT and in 4 APD and 4 PD patients at the third year. 

In patients with the PIGD subtype of parkinsonism, the mean difference between the DAT uptake in the right and left putamens was very small at baseline (Table 5(b)). Therefore, the results in this paper are reported as mean of the right and left sides (except for the relative change in DAT uptake, where the side with the lowest putamen uptake is referred to as the contralateral and the side with the higher putamen uptake is referred to as the ipsilateral side). Accordingly, IBZM uptake in patients is presented as mean of left and right uptake in striatum.

### 2.2. SPECT and Image Evaluation

The SPECT examination timepoints coincided with the clinical evaluation at baseline and reevaluation after 1 and 3 years (HCs were only examined at baseline and after 3 years). The SPECT studies of the patients were retrospectively related to the prospectively updated clinical diagnosis.

All baseline SPECTs in patients were performed in drug naïve state (i.e., no dopaminergic drugs). The follow-up SPECT examinations were made in “pharmacological off” state: ^123^I-FP-Cit SPECT (DAT) was made after a 12 h withdrawal of possible interacting drugs, and the IBZM SPECT was made after a withdrawal of 6 T_1/2_ for each possible interacting drug. Not less than 7 days and not more than 3 months were allowed between the DAT SPECT and the IBZM SPECT at each survey timepoint.

The DAT SPECT was performed 180 minutes after an intravenous bolus dose of 185 MBq of ^123^I-FP-Cit, and the IBZM SPECT was performed 90 minutes after an intravenous bolus dose of 185 MBq of ^123^I-IBZM. For thyroid protection, an oral dose of 200 mg potassium perchlorate was given before and after SPECT. The effective dose for ^123^I-FP-Cit is 4.3 mSv and 6.3 mSv for ^123^I-IBZM. The effective dose from the low dose CT of the head is 0.1 mSv.

#### 2.2.1. Image Acquisition and Reconstruction

The specifications of the two-headed Infinia Hawkeye gamma-camera and the collection of image data were the same as was described in an earlier publication [22]. In summary a low energy general-purpose (LEGP) collimator was used, rendering a spatial resolution (i.e., full width at half maximum) of 9 mm at 10 cm distance from the collimator surface. A 20% energy window was centred on the ^123^I photon energy of 159 keV. Image data were composed in 128 × 128 image matrices with a zoom factor of 1.5, resulting in a pixel size of 2.95 × 2.95 mm, collected by 360 degrees gradual rotation in 120 equally spaced angles for 30 seconds per angle. The CT images were used for the calculation of individual attenuation correction. Ordered subset expectation maximization reconstruction, OSEM (2 iterations, 10 subgroups), was used for reconstruction of the SPECT data. Scatter correction was applied using the triple energy window method, which is the method normally used for this system. The rotation radius was kept as close to 15 cm as possible; however if deviation from 15 cm, the uptake ratio was corrected using a linear equation described in an earlier publication [23] rendering a corrected ratio (in this paper referred to as the “uptake”). 

#### 2.2.2. Image Analysis

Evaluation of the dopamine SPECT examinations of the patients and HC were made blinded to clinical diagnosis and clinical condition. Semiquantitative evaluation of the SPECT images was made by one of the authors (SJM) using an earlier described ROI method using the occipital region as reference [22]. DAT uptake ratios were calculated by dividing the uptake in the striatal regions (caudate, putamen, and whole striatum) with the uptake in the occipital reference region. The IBZM uptake ratios were only calculated for the whole striatum since the low image contrast in the IBZM SPECT images make it difficult to position the relatively small caudate and putamen ROIs with a reliable precision. In HCs the DAT uptake is presented as the average of the uptake ratios in the right and left caudate-, putamen- and striate regions respectively and the IBZM uptake ratio is presented as the average of the ratios in the right and left striate regions.

### 2.3. Statistical Analysis

Continuous data are presented as mean with a 95% confidence interval unless otherwise is stated. Ordinal data is presented as median with minimum and maximum values. For continuous data comparison ANOVA was used with the Bonferroni post-hoc test when comparing more than 2 groups. The Mann Whitney *U* test was used in comparisons of ordinal data, as well as instead of the Student's *t*-test in pairwise comparisons of continuous variables due to small group sizes. Pearson's correlation coefficient was used in correlation analysis to test linear relationships. Change (%) in UPDRS-III scores and DAT and IBZM uptake was calculated as follows: ((value baseline − value 1 year followup)/value baseline) × 100. In HC, the average yearly change (%) in SPECT uptake was estimated by dividing the change (%) between baseline and 3-year follow-up SPECT by 3. *P* values < 0.05 were considered significant, and all *P* values presented are two-sided. Statistical analysis was made using IBM SPSS Statistics 20 (IBM Corp., Armonk, NY, USA).

## 3. Results

### 3.1. Dopamine SPECT Uptake at Different Timepoints

**Figure 2:**
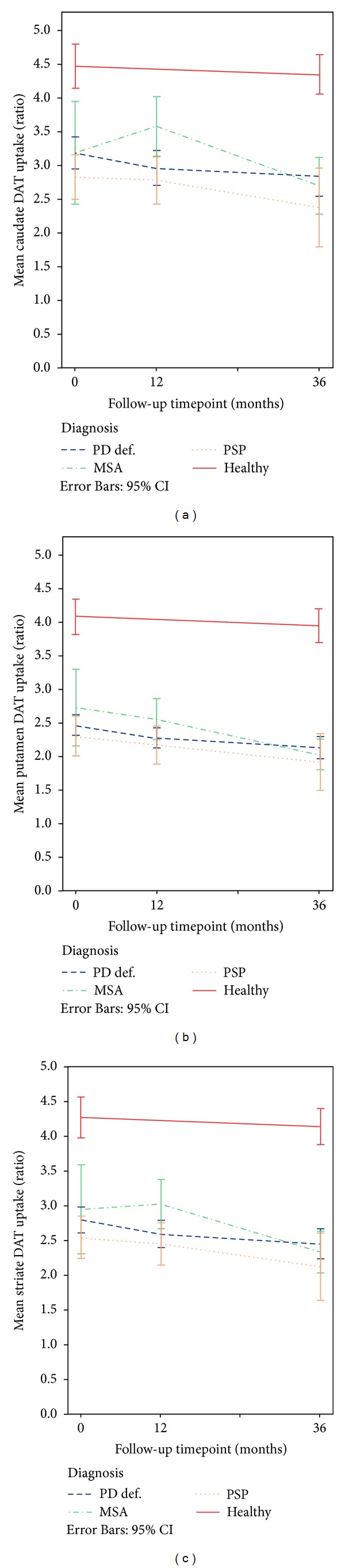
Mean DAT uptake ratios in PD, MSA, and PSP patients and healthy controls in (a) caudate, (b) putamen, (c) striate. Left and right uptake ratios are averaged. Whiskers represent 95% confidence interval of mean. *SPECT: single photon emission computed tomography; DAT: dopamine transporter imaging with* ^123^ *I-FP-Cit; MSA: multiple system atrophy, PSP: progressive supranuclear palsy; PD: Parkinson's disease; Healthy: healthy controls. *

**Figure 3:**
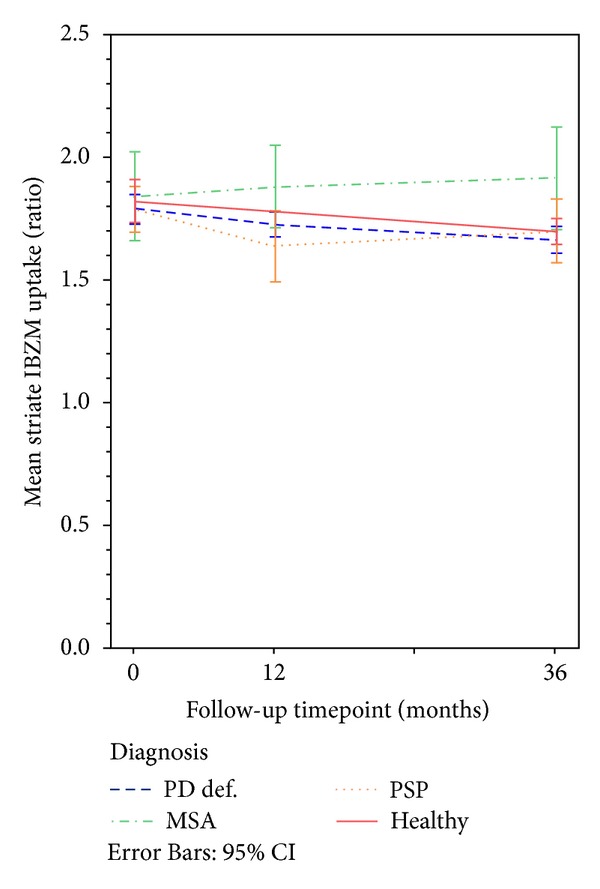
Mean IBZM uptake ratio in PD, MSA, and PSP patients and healthy controls in the striate. Left and right uptake ratios are averaged. Whiskers represent 95% confidence interval of mean. SPECT: single photon emission computed tomography; IBZM: D2/D3 dopamine receptor imaging with  ^123^I-IBZM; MSA: multiple system atrophy, PSP: progressive supranuclear palsy; PD: Parkinson's disease; Healthy: healthy controls.

The mean of right and left DAT and IBZM uptake in patients and HC is presented in Table 6. At baseline, the mean (left and right averaged) DAT uptake was significantly higher in HC compared to patients, *P* < 0.001; however there was no significant difference in DAT uptake between patient groups, that is, PD, MSA, and PSP. The baseline mean IBZM uptake was not significantly different between HC and patients or between patient groups. The mean DAT and IBZM uptake over time in patients and HCs are shown in Figures 2(a), 2(b), 2(c), and 3, respectively.

At the first year followup, mean DAT and IBZM uptake ratios were significantly higher in MSA compared to PSP, *P* < 0.05, and mean DAT caudate and striate uptake were significantly higher in MSA compared to PD, *P* < 0.05. 

The third year, the mean DAT uptake was significantly higher in HC compared to the patients, *P* < 0.001. At this time, no significant differences were seen between the patient groups in DAT uptake. However, MSA patients had significantly higher mean IBZM uptake compared to HC and PD patients, *P* < 0.01 as well as compared to PSP, *P* < 0.05. 

### 3.2. Change in DAT Uptake

In Table 6 the averaged (right and left sides) DAT uptake ratios in each region per SPECT occasion and diagnoses are shown. In PD patients the averaged DAT uptake in putamen was significantly lower after one year compared to baseline, *P* < 0.05 (Mann-Whitney *U* test, asymptotic *P*  value). However, the averaged uptake in the caudate and whole striatum was not significantly different one year after baseline. When comparing the baseline DAT uptake with the uptake at the third year followup in PD patients, the uptake in the putamen was also significantly lower compared to baseline, *P* < 0.01, as well as the DAT uptake in striate, *P* < 0.05. 

There were no significant differences in DAT uptake between baseline and followup after 1 year in MSA or PSP patients. In MSA patients, the averaged putamen uptake as well as the striate DAT was significantly lower at the third year followup compared to baseline, *P* < 0.05. The uptake in both the caudate and putamen as well as the whole striate was also significantly higher at the first year SPECT occasion compared to the third year followup in MSA patients, *P* < 0.05. 

In the PSP patients, the DAT uptake in the putamen at the 3-year follow-up was low compared to baseline; however, the difference did not reach significance. In the group of HCs the mean DAT uptake was not significantly different at baseline compared to 3-year followup.

When considering the whole group of APD patients, the mean DAT uptake in the caudate was significantly lower at the 3-year followup (2.6, 2.31–2.90, and *n* = 10) compared to baseline (2.95, 2.64–3.26, and *n* = 20) *P* < 0.05 as well as the uptake in the putamen (2.0, 1.84–2.16 versus 2.46, and 2.19–2.72), *P* < 0.05 and in the striate (2.27, 2.06–2.48 versus 2.69, and 2.41–2.96), *P* < 0.05. 

### 3.3. Change in IBZM Uptake

In Table 6 the averaged (right and left sides) IBZM uptake ratios in the striate per SPECT occasion and diagnoses are demonstrated. 

No significant change in IBZM uptake was observed after one year compared to baseline in patients with PD, MSA, and PSP. 

When comparing the IBZM uptake at 3-year followup with the uptake at baseline, the IBZM uptake was significantly lower in patients with PD, *P* < 0.01 and in HC, *P* < 0.05. In contrast, in MSA and PSP patients the difference in IBZM uptake between baseline and three years later was not statistically significant.

### 3.4. Relative Change in SPECT Uptake between Baseline and 1-Year Followup

In PD the relative reduction in the striate DAT uptake at the first year followup was significantly more pronounced compared to HC, *P* < 0.01; contralateral caudate, *P* < 0.01, ipsilateral caudate, *P* < 0.05 ipsilateral putamen, *P* < 0.01. However, the reduction rate in the contralateral putamen was not significantly different compared to HC (Table 7). PSP patients also showed a significantly more pronounced reduction rate of DAT uptake in the striatal structures compared to HC, *P* < 0.05. The faster reduction was however not seen in the MSA patients (Table 7). There were no differences in DAT uptake reduction rates between PD and PSP. MSA patients had significantly less relative reduction compared to PD in caudate bilaterally, *P* < 0.05, and in the contralateral caudate and striate compared to PSP patients, *P* < 0.05. There were no significant differences between the relative change in IBZM uptake between HC and patients nor between the patient groups.

### 3.5. SPECT Uptake in Relation to Clinical Parameters

#### 3.5.1. UPDRS-III

At baseline the UPDRS-III score and the DAT uptake were significantly correlated in PD patients (Pearson's correlation coefficients for contralateral caudate: −0.390,  *P* < 0.05 and striate: −0.397, *P* < 0.05; ipsilateral caudate: −0.447, *P* < 0.05, putamen: −0.390, *P* < 0.05, and striate: −0.449,  *P* < 0.05) but no significant correlation was seen in APD patients. The IBZM uptake at baseline did not correlate with the UPDRS-III score, neither in PD nor APD patients. In contrast, at the 1-year followup there was a significant correlation between the UPDRS-III and presynaptic uptake in APD patients (contralateral caudate: −0.526, *P* < 0.05, ipsilateral caudate: −0.598, *P* < 0.01, and ipsilateral striate: −0.507, *P* < 0.05) as well as with the postsynaptic uptake in the contralateral striatum (−0.528, *P* < 0.05), while there was no remaining correlation in PD patients. The change (%) in UPDRS-III score was significantly correlated to the change in presynaptic uptake in APD patients with preserved laterality at 1-year followup (% change in contralateral caudate: −0.604, *P* < 0.05, contralateral striatum: −0.617, *P* < 0.05), but no significant correlation was seen in PD patients. The faster decrease in clinical deterioration seen in the PSP and MSA patients compared to the PD patients probably at least partly reflects the inferior L-Dopa response in PSP and MSA, since the clinical assessment was made in pharmacological “on” state. 

#### 3.5.2. LED

The LED index was significantly correlated to presynaptic uptake in PD patients at baseline (mean caudate: −0.429, *P* < 0.05, mean putamen: −0.389, *P* = 0.04, and mean striate: −0.423, *P* < 0.05) and the same tendency, but without statistical significance, was seen in APD patients. This correlation was not seen in either PD or APD patients at the third year followup. There were no correlations between the IBZM uptake and the LED index at any timepoint. 

#### 3.5.3. Age and Symptom Duration

Age at the time of SPECT was not significantly correlated with either DAT or IBZM uptake, except in PD patients where, at the third year followup, a positive linear relationship was seen between age and the IBZM uptake (Pearson's correlation coefficient: 0.567, *P* < 0.05). In patients with the same laterality as at baseline, the duration of symptoms was correlated to the relative change in presynaptic uptake in APD patients; the longer the disease duration at the 1-year followup SPECT occasion, the less decrease in SPECT uptake between baseline and 1-year followup (contralateral putamen: 0.650, *P* < 0.05, ipsilateral caudate: 0.582, *P* < 0.05, ipsilateral putamen: 0.843, *P* < 0.01, and ipsilateral striate: 0.716, *P* < 0.01).

## 4. Discussion

### 4.1. DAT SPECT Uptake at Baseline and Followup

In this study, we found no significant differences between PD and APS in presynaptic uptake at baseline, which is in line with previous studies. However after one year, PD and PSP patients had significantly lower DAT uptake in contralateral striate compared to MSA patients. After three years the difference was also significant for the ipsilateral striatum. A lower presynaptic uptake in PSP compared to MSA was also seen in a recent positron emission tomography (PET) study using ^18^F-FP-Cit [24]. 

### 4.2. DAT SPECT Uptake Decrease

Typically, the presynaptic reduction rate was higher in patients than in HC but the difference between APS and PD was small. In the present study, most of the PD patients presented with a PIGD subtype and the disease onset was late, factors which are associated with a higher rate of progression compared to early onset and tremor dominant PD [25, 26]. This might also be a reason for the overlapping findings in SPECT uptake between PD and APD in this study. The reduction of the DAT uptake was more rapid in patients compared to HCs. However, a relatively more rapid decline in MSA patients compared to PD claimed in a study using ^123^I-*β*-Cit [16] was not seen in this study. 

The decrease in DAT uptake was most pronounced in PSP patients during the first year, and the decrease slope was similar between the first and third year followup (the difference in DAT uptake ratios between baseline and the third year may not have reached significance due to the large variance at baseline and a small number of PSP patients at the last followup). The mean DAT uptake in especially the caudate in the group of MSA patients tended to increase during the first year after diagnosis. This finding is hard to explain. However, after 3 years the DAT uptake in MSA patients was within levels of the uptake in PD and PSP patients. 

### 4.3. Correlation between Clinical Parameters and DAT SPECT Uptake

The correlation between the UPDRS-III score and presynaptic uptake, seen at baseline in PD patients but not after initiation of medication, is probably explained by the fact that follow-up clinical examination was made in “pharmacological on” state. Supposedly, due to less pharmacological alleviation of symptoms, there was still a correlation between the UPDRS-III score and the DAT uptake in APD patients at followup. The correlation between a longer disease duration and a less decrease in DAT uptake is in line with earlier studies as is reviewed by Brücke et al. [27], explained as that the rate of decline in DAT uptake wears off when the population of remaining dopamine neurons in the substantia nigra reaches a minimum.

### 4.4. IBZM SPECT Uptake at Baseline and Followup

There were no significant differences in IBZM uptake between patients and HC at baseline, nor between HC and PD and PSP patients after 3 years. In the group of MSA patients, the mean IBZM uptake was significantly higher compared to the PD and PSP patients as well as compared to the group of HCs at followup, which will be discussed further in the following.

### 4.5. Change in IBZM SPECT Uptake

A slightly unexpected finding, to our knowledge not reported elsewhere, was that the relative annual decrease rate in IBZM uptake was not significantly different between HCs and patients during the first follow-up year. The HCs in this material are followed clinically and shows no signs of parkinsonism over time and the decline in presynaptic uptake was not excessive, thus probably reflecting an essentially age dependent decline. 

### 4.6. IBZM SPECT Uptake in MSA Patients

In previous studies, APD patients have typically exhibited lower dopamine receptor uptake compared to PD patients [12, 28], but in our study the MSA patients had significantly higher postsynaptic uptake compared HC, PD, and PSP patients at the follow-up SPECT examinations. An upregulation of the D2 receptors due to progression of dopamine depletion has been reported in untreated PD patients [29] and in an experimental PD model [30]; however, to our knowledge this is not reported in MSA, although it is known that not all MSA patients exhibit severe reduction of IBZM uptake [31, 32].

 A persistent relatively high IBZM uptake despite initiated medication with L-Dopa somewhat contrast to the upregulation of postsynaptic receptors seen in untreated PD patients [29] and is difficult to interpret. All MSA cases except one were on pharmacological treatment (with L-Dopa) at both follow-up occasions (however medication was temporarily stopped in all patients prior to the SPECT examination, as specified above), and all except two PSP patients were on dopaminergic treatment. The UPDRS-III, H&Y, and ADL scores indicate a poor response to L-Dopa and progression of clinical symptoms in these MSA patients. L-Dopa response may vary in MSA, and reduced responses have been reported even without histopathological findings of severe striatal D2 receptor depletion [9, 33] suggesting other underlying neuronal mechanisms [34]. One of these might hypothetically be due to receptor-receptor interaction between the adenosine A_2A_ receptors and D2 receptors leading to a reduced affinity to dopamine [35, 36]. This might possibly lead to an upregulation of D2 receptors in remaining medium spiny neurons (MSN) in striatum. Conceivably, the dopamine D2 receptor affinity to the dopamine receptor antagonist IBZM, which is similar to raclopride, might be less impaired [37] thus possible to visualize with SPECT. Another possible reason for persistent upregulation of the dopamine D2 receptors despite dopaminergic treatment with L-Dopa could possibly be an inability to convert L-Dopa into dopamine. In PD the conversion of L-Dopa is thought partly to take place within serotonergic neurons [38, 39]. In MSA, the serotonergic system is affected [40–42], thus this alternative might not contribute to the conversion of L-Dopa. Hypothetically, an inefficient conversion of L-Dopa may result in insufficient net dopamine increase, leaving the remaining dopamine D2 receptors in a hypersensitive state despite medication. The relevance of the above made assumptions is, to our experience, not proposed for MSA elsewhere and may not be applicable to all MSA patients, as the disease exhibit different phenotypes [26, 34]. Furthermore, the group size in this study is small, and the symptomatic disease duration is rather short in the included MSA patients. The degeneration of MSNs in the striatum is progressive [43], thus the uptake of ^123^I-IBZM may decline in later phases of the disease. 

There are some important limitations to this study. First of all, the patient groups are small, and statistics have to be interpreted with caution. The drop-off in the APD group is substantial at the 3-year followup. However, we did not find any obvious systematic bias in the drop-off in this study, since there were no significant differences in clinical parameters between the patients presented here and the group of patients without follow-up SPECT. Secondly, we are aware of the problems with diagnostic accuracy of different forms of idiopathic parkinsonism [2, 44], and although the patients in this study are followed several years, one cannot definitely exclude misdiagnosis in some of the cases. However, we believe that with the long followup of both PD and APD patients in this material as well as the strict use of established diagnostic criteria, the clinical diagnoses are robust, and that our findings in pre- and postsynaptic SPECT uptake are indeed reflecting a panorama of dopamine SPECT findings in early phases of these diagnostic entities. 

## 5. Conclusions

In this study, the discriminative ability of ^123^I-FP-Cit SPECT was high in the first phase of symptomatic and untreated idiopathic parkinsonian disease against healthy individuals in the same age since PD, MSA, and PSP patients all had significantly lower DAT uptake compared to HCs both at baseline and at the followup. The DAT uptake was overlapping between patients with PD, MSA, and PSP; thus ^123^I-FP-Cit SPECT could not reliably separate these diagnostic entities at an early stage of disease. The PSP patients in this study did not have faster decrease in DAT uptake compared to PD patients. However, compared to MSA patients, the decrease rate in PSP during the first year was faster in the contralateral caudate and entire striatum, and the mean DAT uptake in caudate, putamen, and striatum was lower in PSP compared with MSA patients one year after baseline, thus offering a possibility to discriminate between the two diagnoses. 

In this study, the discrimination between PD, MSA, and PSP was not possible with ^123^I-IBZM SPECT in the newly diagnosed, early and untreated stage of clinical disease, since the uptake in PD, MSA and PSP patients was overlapping. Neither were there any difference in IBZM uptake between PD and PSP patients after 1- and 3-year of followup. However, during the follow-up period in this study, the IBZM uptake remained relatively high in the MSA patients despite poor response to medication. In this study, the tendency towards a difference in DAT and IBZM uptake between patients with MSA and PSP was seen over the first years of clinical disease, a finding that may contribute to the discrimination between these diagnoses. 

## Figures and Tables

**Table 1:** The number of SPECTs in patients and HC, per SPECT timepoint.

	SPECT year	PD		APD		HC	
		DAT	IBZM	DAT	IBZM	DAT	IBZM
Number of individuals (m/f)	0	29 (18/11)	29 (18/11)	20 (11/9)	18 (11/7)	14* (6/8)	14* (6/8)
	1	29 (18/11)	29 (18/11)	20 (13/7)	18 (13/5)	0	0
	3	22 (15/7)	17 (11/6)	10 (7/3)	10 (7/3)	16 (7/9)	16 (7/9)

		Mean ± SD	Mean ± SD	Mean ± SD	Mean ± SD	Mean ± SD	Mean ± SD

Age at first symptom		72.9 ± 4.2	72.9 ± 4.2	72.6 ± 8.8	72.2 ± 7.4		
Age at SPECT	0	74.4 ± 4.2	74.5 ± 4.2	74.3 ± 9.2	73.8 ± 9.1	71.5 ± 4.5	71.8 ± 4.7
	1	75.5 ± 4.2	75.5 ± 4.2	75.1 ± 5.1	74.9 ± 5.2		
	3	77.3 ± 4.0	77.8 ± 3.3	76.8 ± 4.4	75.6 ± 4.0	74.3 ± 4.6	74.3 ± 4.5

*Two HCs were scanned on another camera at baseline SPECT: single photon emission computed tomography; DAT: dopamine transporter imaging with ^123^I-FP-Cit; IBZM: D2/D3 dopamine receptor imaging with ^123^I-IBZM; APD: atypical parkinsonian disease; PD: Parkinson's Disease; HC: healthy controls; SD: 1 standard deviation.

**Table 2:** The clinical follow-up time of the patients that underwent DAT SPECT and/or IBZM SPECT at each time-point, per diagnosis.

SPECT year	Diagnosis	*n *	Follow-up time, years
			Mean ± SD
0	PD	29	4.2 ± 1.1
	MSA	7	3.7 ± 1.4
	PSP	13	3.7 ± 1.4
	HC	16	5.0 ± 0.0

1	PD	29	4.2 ± 1.1
	MSA	8	4.1 ± 0.9
	PSP	12	4.2 ± 1.0

3	PD	23	4.3 ± 1.0
	MSA	7	4.9 ± 1.6
	PSP	5	5.1 ± 0.7
	HC	16	5.0 ± 0.0

MSA: multiple system atrophy, PSP: progressive supranuclear palsy; PD: Parkinson's disease. SPECT year 0: baseline, 1: 1 year followup, 3: 3 years followup, and SD: 1 standard deviation.

**Table 3:** MSA and PSP patients with SPECT: diagnoses, gender, subtype, and SPECT per timepoint.

	Gender	Diagnosis	Motor Subtype	SPECT timepoint					
				Baseline		First year		Third year	
				DAT	IBZM	DAT	IBZM	DAT	IBZM
MSA	Male	MSA prob. (+*PD prob.) *	PIGD	∗	∗	∗	∗	X	X
	Female	MSA prob.	PIGD	∗	∗	∗	∗	X	X
	Male	MSA prob.	PIGD	∗	∗	X	X	X	X
	Male	MSA prob. (+*PD prob.) *	PIGD	∗	∗	X	X	X	X
	Male	MSA prob.	PIGD	∗	∗	X	X	X	
	Female	MSA poss. (+*PD def.) *	Indeterm.	X		X	X	X	
	Female	MSA prob.	PIGD	X	X				
	Male	MSA prob.	PIGD	X	X	X	X		
	Female	MSA prob.	PIGD	X	X				
	Male	MSA poss. (+*PD prob.) *	Tremor	X	X	X	X		
	Male	MSA poss. (+*PD def.) *	PIGD	X	X	X	X	X	X
	Male	MSA prob. (+*PD def.) *	Tremor	X	X	X	X		

Total (male/female)		12 (8/4)		7 (4/3)	6 (4/2)	8 (7/1)	8 (7/1)	7 (5/2)	5 (4/1)

PSP	Female	PSP poss.	Indeterm.	∗	∗	X	X		
	Male	PSP poss.	PIGD	∗	∗	X	X		
	Female	PSP prob.	PIGD	∗	∗	X		X	X
	Female	PSP PAGF	PIGD	X	X	X	X		X
	Male	PSP poss.	PIGD	X	X	X	X	X	X
	Male	PSP prob.	PIGD	X	X	X	X	X	X
	Male	PSP poss.	Indeterm.	X	X	X	X		X
	Female	PSP poss. (+*PD def.) *	PIGD	X	X	X			
	Male	PSP poss. (+*PD prob.) *	PIGD	X	X	X	X		
	Female	PSP prob.	PIGD	X	X				
	Male	PSP prob.	PIGD	X	X				
	Female	PSP prob.	PIGD	X	X	X	X		
	Female	PSP prob.	PIGD	X	X	X	X		
	Male	PSP poss.	Tremor	X	X	X	X		
	Male	PSP prob.	PIGD	X	X				
	Female	PSP prob.	PIGD	X					

Total (male/female)		16 (8/8)		13 (7/6)	12 (7/5)	12 (6/6)	10 (6/4)	3 (2/1)	5 (3/2)

The patients fulfilled the established criteria for diagnosis of MSA according to Gilman et al., 1999 [18] or of PSP according to Litvan et al., 1996 [19]. Some patients also fulfilled criteria for PD according toGibb and Lees1988 [20] (diagnosis put in brackets). Field with ∗: SPECT was made on another camera; prob.: probable; PAGF: pure akinesia with gait freezing; poss.: possible; def.: definitive; PIGD: postural instability and gait difficulty; Indeterm.: Indeterminate.

**Table 4:** Clinical data on patients with SPECT shown per SPECT time-point.

		PD				MSA				PSP			
	SPECT year	DAT		IBZM		DAT		IBZM		DAT		IBZM	
		*n* = 29/29/22		*n* = 29/29/17		*n* = 7/8/7		*n* = 6/8/5		*n* = 13/12/3		*n* = 12/10/5	
		Mean	95% CI	Mean	95% CI	Mean	95% CI	Mean	95% CI	Mean	95% CI	Mean	95% CI
Age	0	74.4	72.8–76.0	74.5	72.9–76.1	71.3	60.7–81.9	69.9	57.4–82.3	75.9	71.3–80.6	75.3	70.4–80.2
	1	75.5	73.9–77.1	75.5	73.9–77.1	76.3	73.3–79.2	76.2	73.4–79.0	74.3	70.5–78.1	73.8	69.2–78.5
	3	77.3	75.5–79.1	77.5	75.8–79.3	78.7	75.9–81.5	77.5	74.7–80.3	72.5	61–84	75.9	68.1–83.7
Duration	0	1.5	1.2–1.8	1.6	1.2–1.9	1.3	0.6–2.0	1.5	0.7–2.3	1.9	1.1–2.7	1.8	1.0–2.7
	1	2.6	2.3–3.0	2.6	2.3–3.0	3.1	2.2–4.0	3.0	2.1–3.9	2.9	1.9–3.9	2.8	1.6–4.0
	3	4.5	4.1–4.9	4.4	3.9–4.8	5.1	4.1–6.2	5.5	4.1–6.8	4.6	0.3–9.0	5.0	3.1–6.9
LED	0	0.0	0.0–0.0	0.0	0.0–0.0	0.0	0.0–0.0	0.0	0.0–0.0	0.0	0.0–0.0	0.0	0.0–0.0
	1	335	273–397	335	273–397	312	160–465	312	160–465	340	157–522	287	108–467
	3	507.4	419–595	439	352–526	582	404–761	620	398–842	708	202–1214	515	92–938

		Median	Min–Max	Median	Min–Max	Median	Min–Max	Median	Min–Max	Median	Min–Max	Median	Min–Max

UPDRS-III	0	27	8–46	27	8–46	22	9–48	22.5	21–48	30	12–64	29	12–64
	1	25	3–48	25	3–48	24	14–44	24	14–44	36^†^	18–54	35.5^††^	18–54
	3	25	3–48	23	6–54	41*	31–65	41.0*	33–49	48^†^	43–55^†^	43^††^	38–55

UPDRS% Change during first year		(*n* = 29) Median −7.7 Min −87–Max 52.9				(*n* = 8) Median 8.9* Min −22.5–Max 55.6				(*n* = 11) Median 42.3^††^ Min 2.4–Max 77.8			

H&Y	0	2	1–3	2	1–3	2	2–5	2	2–5	2.5	1.5–5	2.5	1.5–5
	1	2	1–3	2	1–3	2	2–3	2	2–3	2.75^†^	1.5–5	2.75^††^	2–5
	3	2	2–4	2	2–3	3*	2–5	3*	2–4	4^†^	2–5	2.5^†^	2–5
ADL%	0	90	60–95	90	60–95	90	60–100	90	60–100	80	70–95	80	70–95
	1	90	60–95	90	60–95	90	70–90	90	70–90	80^†^	50–95	80^††^	50–95
	3	90	40–100	90	40–100	80*	40–80	80*	70–80	70	30–90	70	30–90

Mann-Whitney *U* test: *PD versus MSA: *P* < 0.05; ^†^PD versus PSP: *P* < 0.05; ^††^PD versus PSP: *P* < 0.01; ^†††^PD versus PSP: *P* < 0.001. year: 0: baseline, 1: first year, 3: third year; LED: L-Dopa equivalent dose index; UPDRS-III: Unified Parkinson's Disease Rating Scale, motor examination; H&Y: Hoehn and Yahr stage; ADL: Activities of daily living scale; 95% CI: 95% confidence interval.

**Table:** (a)

	Clinical laterality at baseline				
	No reported laterality	Left	Right	Symmetrical	Total
Side with lowest DAT uptake in putamen					
Right	1	11	3	11	*26 *
Left	5	0	10	8	*23 *
Total	*6 *	*11 *	*13 *	*19 *	*49 *
Parkinsonism type					
PIGD	6	5	6	15	*32 *
Tremor	0	1	6	3	*10 *
Indeterminate	0	5	1	1	*7 *
Total	*6 *	*11 *	*13 *	*19 *	*49 *

PIGD: postural instability and gait difficulty and 95% CI: 95% confidence interval.

**Table:** (b)

Parkinsonism motor subtype	*n *	Mean left/right ratio	95% CI
PIGD	32	1.00	0.95–1.05
Tremor	10	0.93	0.82–1.03
Indeterminate	7	1.15	0.99–1.30
Total	*49 *	*1.01 *	*0.96–1.05 *

PIGD: postural instability and gait difficulty and 95% CI: 95% confidence interval.

**Table:** (a)

Baseline														
	HC DAT *n* = 14 IBZM *n* = 14		PD DAT *n* = 29 IBZM *n* = 29			MSA DAT *n* = 7 IBZM *n* = 6			PSP DAT *n* = 13 IBZM *n* = 12			Mann-Whitney *U* test		
	Mean	95% CI	Mean	95% CI	PD versus HC	Mean	95% CI	MSA versus HC	Mean	95% CI	PSP versus HC	PD versus MSA	PD versus PSP	MSA versus PSP
					P*			P*			P*	*P *	*P *	*P *
Caudate DAT	4.47	4.14–4.80	3.18	2.94–3.42	<0.001	3.19	2.43–3.95	<0.001	2.83	2.50–3.15	<0.001	ns	ns	(0.075)
Putamen DAT	4.09	3.83–4.36	2.47	2.32–2.63	<0.001	2.74	2.16–3.31	<0.001	2.31	2.01–2.61	<0.001	ns	ns	(0.075)
Striate DAT	4.27	3.98–4.56	2.80	2.61–2.98	<0.001	2.95	2.31–3.59	<0.001	2.55	2.25–2.85	<0.001	ns	ns	(0.063)

Striate IBZM	1.82	1.74–1.91	1.79	1.73–1.85	1.00	1.84	1.66–2.03	1.00	1.79	1.70–1.88	1.00	ns	ns	ns

**Table:** (b)

Year 1									
	PD DAT *n* = 29 IBZM *n* = 29		MSA DAT *n* = 8 IBZM *n* = 8		PSP DAT *n* = 12 IBZM *n* = 10		Mann-Whitney *U* test		
	Mean	95% CI	Mean	95% CI	Mean	95% CI	PD versus MSA	PD versus PSP	MSA versus PSP
							*P *	*P *	*P*
Caudate DAT	2.96	2.70–3.22	3.58	3.13–4.02	2.78	2.43–3.13	0.024	ns	0.007
Putamen DAT	2.28	2.13–2.43	2.56	2.25–2.87	2.17	1.89–2.46	(0.060)	ns	0.025
Striate DAT	2.59	2.40–2.79	3.02	2.67–3.38	2.45	2.15–2.76	0.029	ns	0.014

Striate IBZM	1.73	1.67–1.78	1.88	1.71–2.05	1.64	1.49–1.78	(0.051)	ns	0.026

**Table:** (c)

Year 3														
	HC DAT *n* = 16 IBZM *n* = 16		PD DAT *n* = 22 IBZM *n* = 17			MSA DAT *n* = 7 IBZM *n* = 5			PSP DAT *n* = 3 IBZM *n* = 5			Mann-Whitney *U* test		
	Mean	95% CI	Mean	95% CI	PD versus HC	Mean	95% CI	MSA versus HC	Mean	95% CI	PSP versus HC	PD versus MSA	PD versus PSP	MSA versus PSP
					P*			P*			P*	*P *	*P *	*P *
Caudate DAT	4.35	4.06–4.63	2.83	2.55–3.12	<0.001	2.70	2.28–3.12	<0.001	2.38	1.80–2.96	<0.001	ns	ns	ns
Putamen DAT	3.96	3.71–4.21	2.13	1.97–2.30	<0.001	2.03	1.80–2.26	<0.001	1.92	1.50–2.34	<0.001	ns	ns	ns
Striate DAT	4.14	3.88–4.40	2.45	2.24–2.67	<0.001	2.33	2.03–2.63	<0.001	2.12	1.64–2.61	<0.001	ns	ns	ns

Striate IBZM	1.70	1.65–1.75	1.66	1.61–1.72	1.0	1.92	1.71–2.13	0.003	1.70	1.57–1.83	1.00	0.002	ns	0.016

Left and right uptake ratios are averaged.*One way ANOVA, Bonferroni post-hoc test *P* value. 95% CI: 95% confidence interval.

**Table 7:** Yearly change (%) in uptake between baseline and 1-year followup: DAT and IBZM ratios.

	*N *	Mean	95% CI	Mann-Whitney *U* test					
				HC versus PD	HC versus MSA	HC versus PSP	PD versus MSA	PD versus PSP	MSA versus PSP
				*P *	*P *	*P *	*P *	*P *	*P *
Contralateral^∞^									
** **Caudate DAT									
PD	27	−8.0	−11.6–−4.5	0.005	ns	0.006	0.013	ns	0.017
MSA	4	5.3	−4.8–15.4						
PSP	8	−11.8	−21.0–−2.7						
HC*	14	−0.5	−2.2–1.3						
** **Putamen DAT									
PD	27	−6.5	−10.8–−2.2	ns	ns	0.006	ns	ns	ns
MSA	4	−3.3	−14.4–7.8						
PSP	8	−13.9	−23.0–−4.7						
HC*	13	−1.0	−2.9–0.8						
** **Striate DAT									
PD	27	−7.2	−10.6–−3.9	0.006	ns	0.004	ns	ns	0.042
MSA	4	0.8	−10.4–11.9						
PSP	8	−12.8	−21.6–−4.0						
HC*	14	−0.7	−2.4–0.9						
Ipsilateral^∞^									
** **Caudate DAT									
PD	27	−7.7	−12.1–−3.4	0.045	ns	0.006	0.045	ns	ns
MSA	4	4.3	−12.3–21.0						
PSP	8	−11.9	−24.8–1.1						
HC*	14	−0.5	−2.2–1.3						
** **Putamen DAT									
PD	27	−8.5	−12.0–−4.9	0.006	ns	0.005	ns	(0.054)	ns
MSA	4	−8.2	−28.0–11.5						
PSP	8	−13.4	−26.9–0.1						
HC*	13	−1.0	−2.9–0.8						
** **Striate DAT									
PD	27	−8.3	−11.7–−5.0	0.002	ns	0.004	ns	ns	ns
MSA	4	−1.9	−19.2–15.4						
PSP	8	−12.8	−25.5–0.0						
HC*	14	−0.7	−2.4–0.9						

Mean									
** **Striate IBZM									
PD	29	−3.1	−6.2–0.0	ns	ns	ns	ns	ns	ns
MSA	5	−4.7	−8.7–−0.7						
PSP	8	−8.3	−19.8–3.3						
HC*	14	−2.1	−3.5–−0.8						

*HC: average yearly change calculated between baseline and the 3-year follow-up SPECT.

^∞^Patients with same laterality as baseline.
